# Development of a survey instrument to investigate the primary care factors related to differences in cancer diagnosis between international jurisdictions

**DOI:** 10.1186/1471-2296-15-122

**Published:** 2014-06-17

**Authors:** Peter W Rose, Willie Hamilton, Kate Aldersey, Andriana Barisic, Martin Dawes, Catherine Foot, Eva Grunfeld, Nigel Hart, Richard D Neal, Marie Pirotta, Jeffrey Sisler, Hans Thulesius, Peter Vedsted, Jane Young, Greg Rubin

**Affiliations:** 1 Department of Primary Care Health Sciences, New Radcliffe House, 2nd Floor, Radcliffe Observatory Quarter, University of Oxford, Woodstock Road, Oxford OX2 6GG, UK; 2 University of Exeter Medical School, College House, St Luke’s Campus, Magdalen Road, Exeter EX1 2LU, UK; 3 ICBP Programme, Cancer Research UK, Angel Building, 407 St John Street, London EC1V 4AD, UK; 4 Department of Prevention and Cancer Control, Cancer Care Ontario, 620 University Avenue, Toronto, ON M5G 2L7, Canada; 5 Department of Family Practice, David Strangway Building, University of British Columbia, 5950 University Boulevard, Vancouver, BC V6T 1Z3, Canada; 6 The King’s Fund, 11–13 Cavendish Square, London W1G 0AN, UK; 7 Knowledge Translation Research Network Health Services Research Program, Ontario Institute for Cancer Research, Toronto, ON, Canada; 8 Department of Family and Community Medicine, University of Toronto, 500 University Avenue, Toronto, ON M5G 1V7, Canada; 9 School of Medicine, Dentistry and Biomedical Sciences - Centre for Public Health, Queen’s University Belfast 2013, University Road, Belfast BT7 1NN, UK; 10 Primary Care Medicine, North Wales Centre for Primary Care Research, Bangor University, Gwenfro Units 4-8, Wrexham Technology Park, Wrexham LL13 7YP, UK; 11 Primary Health Care Research Evaluation and Development, Department of General Practice, 200 Berkeley Street, Carlton, Victoria 3053, Australia; 12 Division of Continuing Professional Development, Department of Family Medicine, University of Manitoba, 727 McDermot Avenue, Winnipeg, Manitoba R3E 3P5, Canada; 13 Lund University, Box 117, SE-221 00 Lund, Sweden; 14 Research Unit for General Practice, Department of Public Health, Aarhus University, Bartholins Allé 2, 8000, Aarhus C, Denmark; 15 Cancer Epidemiology, Public Health, School of Public Health, D02-QE11 Research Institute for Mothers and Infants, The University of Sydney, Sydney 2006, Australia; 16 Wolfson Research Institute, Queen’s Campus, Durham University, Stockton on Tees TS17 6BH, UK

**Keywords:** Survey, Primary care, Cancer, Diagnosis, International

## Abstract

**Background:**

Survival rates following a diagnosis of cancer vary between countries. The International Cancer Benchmarking Partnership (ICBP), a collaboration between six countries with primary care led health services, was set up in 2009 to investigate the causes of these differences. Module 3 of this collaboration hypothesised that an association exists between the readiness of primary care physicians (PCP) to investigate for cancer – the ‘threshold’ risk level at which they investigate or refer to a specialist for consideration of possible cancer – and survival for that cancer (lung, colorectal and ovarian). We describe the development of an international survey instrument to test this hypothesis.

**Methods:**

The work was led by an academic steering group in England. They agreed that an online survey was the most pragmatic way of identifying differences between the jurisdictions. Research questions were identified through clinical experience and expert knowledge of the relevant literature.

A survey comprising a set of direct questions and five clinical scenarios was developed to investigate the hypothesis. The survey content was discussed and refined concurrently and repeatedly with international partners. The survey was validated using an iterative process in England. Following validation the survey was adapted to be relevant to the health systems operating in other jurisdictions and translated into Danish, Norwegian and Swedish, and into Canadian and Australian English.

**Results:**

This work has produced a survey with face, content and cross cultural validity that will be circulated in all six countries. It could also form a benchmark for similar surveys in countries with similar health care systems.

**Conclusions:**

The vignettes could also be used as educational resources. This study is likely to impact on healthcare policy and practice in participating countries.

## Background

There are acknowledged differences in cancer survival rates between countries with similar, primary health care led health systems [1]. The International Cancer Benchmarking Partnership (ICBP) was established with the aims of producing up-to-date survival estimates for selected cancers (breast, colorectal, lung, ovary), establishing whether these differences have changed over time and particularly to investigate possible causes of survival deficits identified [2]. It comprises five work streams, one of which (Module 3) is focused on primary care aspects of cancer diagnosis. Specifically, this aspect relates to the period between the patient’s first presentation to a primary care practitioner (PCP) with a symptom of possible oncological significance up to the time that a referral is made to secondary care for further diagnostic investigation or for treatment, when the diagnosis of cancer is made in primary care.

There is increasing evidence that the time from first presentation of cancer to diagnosis is associated with prognosis [3-5]. The aim of Module 3 was to identify differences in primary care systems, structure or clinical practice that might contribute to known differences in cancer outcomes between ICBP jurisdictions. Specifically this related to factors that might influence delay in diagnosis or referral within primary care [6]. Such factors can be: 1) structural, such as access to investigations, access to specialist advice, 2) organisational, such as degree of gatekeeping [7] and safety netting practices, and 3) knowledge and skills, such as the awareness of cancer symptoms and diagnostic skills among PCP.

In order to undertake such a study, we needed a valid and reliable measure of the differences in awareness, skills, structure and organisation between different primary care settings. The aim of this paper is to present the development of this measurement tool and the challenges that had to be addressed in the design and conduct of a survey of ICBP jurisdictions. The eleven jurisdictions, located in six countries, were England, Northern Ireland, Wales, Denmark, Norway, Sweden, British Columbia, Manitoba, Ontario, New South Wales and Victoria. Each jurisdiction contributed to the costs of the project and necessary ethical approvals.

## Methods

### Conceptualisation

**Figure 1:**
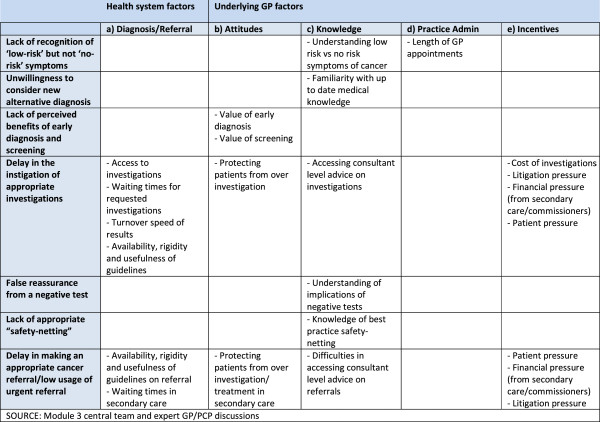
Systematic, organisational and clinical factors within primary care which could contribute to international differences in cancer diagnosis.

A group of primary care practitioners with expertise of cancer diagnosis, drawing on input from a review of literature, clinical experience and advice from a group of three international experts in the field, identified features and aspects of primary care systems, organisation and clinical practice which could contribute to international differences in the diagnosis of cancer [8-15]. From this a set of hypotheses was generated for testing (Figure 1). Features of primary care practice which were hypothesised as being influential included health system factors, diagnostic factors and referral factors as well as factors related to PCP behaviours, attitudes, skills, knowledge, practice administration and incentives. Initially, screening was also included but was then removed as the primary aim was in explaining differences in symptomatic diagnosis. The process was iterative, starting with all factors that could be relevant and then reducing these based on perceived importance, relevance to all jurisdictions, and feasibility for testing in a survey format. Decisions were made on a consensus basis until there was agreement on the form, structure and content of the survey to fully investigate the hypothesis. Teleconferences were scheduled at regular intervals and active email communication conducted to facilitate decision making at all stages of the study.The features that were hypothesised to be important in understanding and evaluating differences between countries were grouped into two main categories: general structural and cultural factors; and specific clinical, educational and organisational aspects. We chose to focus on the features related to the individual PCP and their activity in relation to cancer diagnosis. An international collaboration ethics approval was sought as required in each jurisdiction (see Figure 1).

To capture the differences in the generic aspects of health care systems, the ICBP Programme Board commissioned a comparative analysis of health care systems to contextualise the results of this study. This ‘system mapping’ exercise represents a comparison of the health care systems found in each jurisdiction relating to cancer diagnosis, is reported elsewhere (Brown S, Rubin G, Castelli M, Hunter DJ, Erskine J, Vedsted P, Foot C: How might health care systems influence speed of cancer diagnosis: a narrative review, in preparation).

### Key hypotheses of causes of delays to cancer diagnosis in primary care

To ensure content validity was present for all jurisdictions, all features were discussed iteratively. Recognising that long surveys affect response and completion rates, consensus was reached between the collaborators on the features considered to be the most important (Figure 1).

### Choice of data collection method

A number of methodologies were considered including a questionnaire survey, system mapping, primary care notes review, simulated cases, qualitative interviews or focus groups with PCPs. We opted for survey methodology delivered electronically, as the most easily reproducible in several countries and languages, the easiest in which to maintain consistency between countries and its reasonable cost, whilst accessing the views of a larger number of PCPs.

### Operationalisation and development of survey

The survey was developed in two parts. The first part consisted of five clinical vignettes to capture the aspects of recognition of ‘low-risk but not no-risk’ symptoms, delay in instigating investigations and reluctance to consider an alternative diagnosis. Vignettes are recognised to produce a better assessment of quality of care compared to record audit and they are faster to perform and more economical [16]. Vignettes also predict physician performance as judged against consultations with trained actors and can be a good measure of process of care [17]. They have also been validated in electronic form and used to measure care across different health systems in California [18].

The vignettes were based on common clinical presentations of possible lung (two vignettes), colorectal (two vignettes) and ovarian (one vignette) cancers. They were evidence-based, using primary care evidence on symptoms/sign and positive predictive values [19-22]. Breast cancer was omitted as we considered it very likely all women with a breast lump would be investigated, and there is very little primary care evidence to support investigation/non-investigation of other breast symptoms.

Each vignette was presented in two or three “phases”, with the second and third phase of each vignette representing a further presentation of the patient with additional symptoms or worsening severity of initial symptoms. Respondents were asked questions about management of the cases, using a drop-down menu for responses. The vignette ended if the respondent decided to refer the patient to hospital or undertake a test themselves that would confirm a diagnosis of cancer if present (chest xray or lung CT for lung vignettes, colonoscopy or abdominal CT for colorectal vignettes, abdominal CT or abdominal or trans-vaginal ultrasound for the ovarian vignette). We labelled these ‘near-definitive’ tests, while accepting each has a (small) false-negative and false-positive rate. Other common primary care tests, such as haemoglobin or tumour markers have considerably less predictive accuracy, so if a respondent chose to perform one of these, the vignette continued. At the end of the vignette the respondent was given a diagnosis for the patient in the vignette. The final outcome in three vignettes (2, 4 and 5) was cancer. In two vignettes (1 and 3) the final diagnosis was not cancer (i.e. bronchiectasis, symptoms cleared up). This was done to reduce the bias inherent in assessing clinical performance when respondents were aware the survey related to cancer diagnosis. The assessment was based on the management of each vignette and the final outcome was not relevant to this.

The second part of the survey consisted of direct questions addressing aspects of the responder’s local health care system and their own attitudes and education.

Simple demographic data relating to gender, type of PCP, time since qualification, location of training and rurality of practice were also identified.

Collaborators in all jurisdictions agreed to develop a core survey relevant to all, but to allow individual jurisdictions to add a small number of additional locally relevant questions at the end of the survey. These additional questions were subject to approval based on the overall length of the survey being acceptable to the central research team.

### Overseeing the instrument development

At every stage the development of the instrument was discussed with the ICBP Programme Board and the Module 3 leads from each participating jurisdiction. The challenges of ensuring participation in teleconferences across disparate time zones was successfully addressed by holding teleconferences with identical agendas at two different times in the same day, with the chair and programme management team present at both to provide continuity.

### Validation

At every stage, the survey was discussed with each jurisdiction to confirm that features being assessed were relevant to the hypotheses, whilst remaining locally cogent. During this process, some questions were omitted due to lack of international applicability. These included the relevance and use of guidelines which varied between jurisdictions, issues of differential care to remote communities (relevant to Canada, Australia and Norway), variations in care between publicly and privately insured patients, and questions related to screening of asymptomatic patients. Each of these factors was seen to have particular local pertinence, but less international relevance. These were topics taken up by some jurisdictions that asked additional (non-core) questions in their local survey. Thus, content validity of the aspects was ensured during the conceptualisation.

The face validity of the final items was tested twice. The initial survey was piloted by four English academic PCPs who were asked to complete a paper-based version of the survey and give written and verbal feedback on the relevance of items, whether the items covered the area of interest and whether they would be able to interpret results from the answers. They were also asked about their understanding of the questions. Amendments were made in the light of their feedback to develop a second draft.

The second draft was tested out on seven English PCPs of varying age, gender and background. Six were seen face to face and the seventh provided written feedback. The PCPs were asked to complete a prototype electronic version of the survey in the presence of the researcher, who then used a cognitive interviewing technique to ascertain the relevance of the survey and its content, including understanding of the meaning of the questions involved. The PCPs considered instructions to be appropriate, the content was relevant and the vignettes represented clinical cases that they could recognise from their clinical practice [16]. They were clear that the purpose of the vignettes was not a ‘test’ of their practice, but to identify how they would manage a patient’s symptoms. Suggestions were made to clarify the meaning of some items.

Feasibility was tested on both of these occasions with all PCPs reporting that the time to complete the survey was reasonable; all completed the survey within 20 minutes.

## Results

### Testing consistency

In the vignettes the respondents were asked how they would manage each case, including which investigations they would undertake in primary care. Respondents were asked to choose only tests that they had direct access to (tests that could be ordered by the GP without reference to a specialist doctor) in their own practice. In the direct question section, respondents were also asked which tests were available by direct access in their jurisdiction. As a measure of consistency, we measured how many respondents ordered tests in the vignettes that were not available to them through direct access, according to their response to the question on this point. This cross-validation exercise was performed on the first jurisdiction to complete the survey (Denmark). We identified and assessed the use of those tests where at least 80% of respondents did not have direct access to the tests: CT/MRI of lung for vignettes one and two, CT abdomen for vignettes three, four and five and colonoscopy for vignettes three and four. The percentages of these Danish respondents who ordered these tests despite stating no access were low: vignette one = 9%; vignette two = 4.2%; vignette three, CT abdomen = 0%, colonoscopy = 0%; vignette four, CT abdomen = 2.9%, colonoscopy = 0%; vignette five, CT abdomen = 5.3%.

We did not determine test-retest reliability because we predicted that answers would differ over time. This would be especially true of the vignettes as respondents were told the outcome of each vignette at the end of the survey.

### Translation and adaptation

The final English version of the survey was adapted in the other English-speaking countries outside the UK. For the Canadian and Australian jurisdictions, there was an adaptation of certain specific terms to improve sense in these jurisdictions (such as ‘office’ or ‘clinic’ instead of ‘practice’). Together with the collaborators in Canada it was agreed not to make a translation into Canadian French and with collaborators in Wales not to make a translation in Welsh.

The final validated UK English version of the survey was translated into Danish, Norwegian and Swedish, following methods already described [23]. To take advantage of the commonalities between the Scandinavian languages, the survey was first translated into Danish. The translation into Danish followed a standardised process [23]. Translations were done by two native Danish speakers who spoke good English (one professionally in English medical language and one an English correspondent). The translation was then checked by two Danish PCPs and any problems discussed with the translators at an expert meeting. Then there was a back-translation into English made by two English native speakers who also spoke Danish fluently. Both were familiar with medical terminology. The back-translation was compared and discussed and semantic differences with the original version were discussed at a second expert meeting. We aimed for conceptual and cultural equivalence rather than a verbatim translation. Items were culturally adapted to reflect the Danish healthcare systems. Discussion between the central team and Danish collaborators was then undertaken to check equivalence of linguistic, cultural and professional meaning with the UK English version.

The final Danish version was then pilot tested among four PCPs before being translated into Swedish and Norwegian. These translations were made by a single translation into Swedish and Norwegian, respectively. These versions were not back-translated. The final Norwegian and Swedish versions were culturally and structurally adapted. The Swedish version was also tested on 20 PCPs and registrars.

### Pilot testing of the final version

The survey was converted into an electronic version by a commercial company (Sigmer Technologies Limited). The electronic version was then tested by 16 PCPs in the UK. No issues were identified concerning how the electronic version of the survey worked. However, the time taken to complete all five vignettes was considered to be too long and the central team decided to ask each respondent to answer only two vignettes each. These vignettes were assigned randomly, with each referring to a different cancer, with either a positive cancer diagnosis followed by a negative vignette or *vice versa*. Respondents knew this was a cancer related survey, so the choice and outcome of vignettes was randomized to minimise bias.

### Sample selection

Each jurisdiction decided on a method of sampling and approach to potential participants (regular post or email), dependant on local conditions and the availability of databases with PCP contact details. Participants were PCPs in regular day time primary care, locums or those working in ‘out-of-hours’ services. Retired PCPs and those in training were not eligible, and other primary care providers such as nurse practitioners were not included.

### Sample size

Each jurisdiction was expected to recruit at least 200 respondents. A sample size of 200 has a 95% confidence interval (CI) of 43-57% for an equally distributed response (50% respond ‘yes’), and a CI of 15-26% for a response where 20% of respondents say ‘yes’.

### Analysis plan

The answers to direct questions will be presented as simple descriptive statistics. This will enable comparison between jurisdictions of several stages of the process from presentation of cancer symptoms to diagnosis in primary care or referral. This includes length of consultations, safety-netting practices (processes to ensure appropriate patient follow-up), availability and wait for tests and test results, availability of advice and speed of referral to first appointment.

The main outcome of interest in the vignettes will be the proportion of respondents in each jurisdiction who ‘completed’ the vignette (i.e. made a referral or undertook a definitive diagnostic test) at each stage compared to the one-year survival and five-year [conditional on one year] survival for that cancer in a given jurisdiction [1,24]. Both of these survival outcomes are affected by factors in the period before referral to hospital. The conditional 5 year survival (i.e. 5 year survival conditional on surviving at least 1 year) minimises the impact of factors that primarily affect survival in the first year after diagnosis, such as delays in diagnosis and aggressiveness of the tumour. Regression analyses will seek associations between other factors investigated in the survey and survival rates.

### The final survey

Copies of the survey are available upon request from the ICBP programme management team at icbp@cancer.org.uk.

## Discussion

This paper describes the development of a survey to assess the differences in primary care as it relates to cancer diagnosis amongst 11 jurisdictions (England, Northern Ireland, Wales, Denmark, Norway, Sweden, British Columbia, Manitoba, Ontario, New South Wales and Victoria) that make up part of the ICBP. The purpose of the survey is to identify and understand differences in primary care systems and in the clinical practice of PCPs that might explain the differences in cancer outcomes between these jurisdictions. The survey was tested extensively before completion, including checks to ensure cross-cultural validity.

Other surveys have used similar methodology, especially relating to the use of vignettes and this method has good correlation with clinical practice [18]. There are no similar surveys investigating the diagnosis of cancer across a large number of jurisdictions. The survey is relevant to clinical practice in countries with a primary care led health service and contains clinical situations that are familiar to PCPs. The electronic nature of the survey makes it possible to use vignettes with multiple options. It is easily accessible and easy to conduct and it will provide strong comparative data as a result. Its use would be restricted in countries with limited internet access for PCPs.

Iterative testing of the survey was undertaken, both in England where the survey was developed but also in some other jurisdictions to ensure face validity, content validity and cross cultural validity. Extensive piloting among all jurisdictions was limited by the need to develop the survey at the same time as jurisdictions were being recruited and adapting the survey for local use. More extensive pilot testing was also limited by constraints of time and resources. Reliability testing was consequently difficult in the pilot stage due to small numbers of respondents in the pilot stages, but testing of consistency in the early stage of the actual survey showed a high level of consistency in the vignettes with the exception of the use of CT lung scans in the lung cancer vignettes; 9% of respondents ordering a lung CT to investigate the cases had stated that they did not have access to this test. However, even in these vignettes consistency scores were considered acceptable.

Restricting the survey to only two of five possible vignettes might affect validity of results by reducing the sample size for each vignette. However, this was felt necessary to ensure survey completion time was reasonable and to enable exploration of other issues not amenable to the use of vignettes, including structural and organisational factors.

The survey will have future value in providing a benchmark against which other studies could be measured and in providing a ‘template’ that could be adjusted to local circumstances for similar studies to be undertaken in other *settings*.

## Conclusions

We have developed and validated a survey instrument that investigates the diagnosis of cancer by primary care physicians. We intend to use the instrument to compare current practice between six countries whose health services are led by primary care. Other countries with similar health systems could use this study as a benchmark and the survey could be repeated to identify changes with time. The vignette part of the survey could also be used as an educational tool.

We anticipate that the findings from ICBP Module 3 will have an impact on healthcare policy and practice in the participating jurisdictions and begin to indicate primary care factors that could impact on survival differences between participating jurisdictions.

## Appendix A – Working Group

Andriana Barisic, Research Associate, Department of Prevention and Cancer Control, Cancer Care Ontario, 620 University Avenue, Toronto, Ontario, M5G 2 L7, Canada.

Martin Dawes, Head, Department of Family Practice, David Strangway Building, University of British Columbia, 5950 University Boulevard, Vancouver, British Columbia, V6T 1Z3, Canada.

Diana Dawes, Research Associate, Department of Family Practice, David Strangway Building, University of British Columbia, 5950 University Boulevard, Vancouver, British Columbia, V6T 1Z3, Canada.

Mark Elwood, Vice-President, Family and Community Oncology, BC Cancer Agency; Clinical Professor, School of Population and Public Health, UBC; Honorary Professor, Department of Epidemiology and Preventive Medicine, Monash University, Melbourne, Australia.

Kirsty Forsdike, Senior Research Assistant, Department of General Practice, 200 Berkeley Street, Carlton Victoria 3053, Australia.

Eva Grunfeld, Director, Knowledge Translation Research Network Health Services Research Program, Ontario Institute for Cancer Research; Professor and Vice Chair Research Department of Family and Community Medicine, University of Toronto, 500 University Avenue, Toronto, Ontario, M5G 1 V7, Canada.

Nigel Hart, Clinical Senior Lecturer, School of Medicine, Dentistry and Biomedical Sciences - Centre for Public Health, Queen’s University Belfast 2013, University Road Belfast, BT7 1NN, United Kingdom.

Breann Hawryluk, Project Planning Coordinator, Department of Patient Navigation, Cancer Care Manitoba, 675 McDermot Street, Winnipeg, Manitoba, Canada.

Gerald Konrad, Associate Professor, Department of Family Medicine, University of Manitoba 5–400 Tache Avenue, Winnipeg, Manitoba, Canada.

Anne Kari Knudsen, Administrative leader, Department of Cancer Research and Molecular Medicine, Norwegian University of Science and Technology, 7489 Trondheim.

Magdalena Lagerlund, Department of Learning, Informatics, Management and Ethics, Karolinska Institute, Berzelius väg 3, Stockholm 171 77, Sweden.

Claire McAulay, Research Officer, Public Health, School of Public Health, D02-QE11 Research Institute for Mothers and Infants, University of Sydney NSW 2006 Australia.

Jin Mou, Postdoctoral Fellow, Department of Family Practice, Research Office, Department of Family Practice, David Strangway Building, University of British Columbia, 5950 University Boulevard, Vancouver, British Columbia, V6T 1Z3, Canada.

Richard D Neal, Professor of Primary Care Medicine and Director, North Wales Centre for Primary Care Research, Bangor University, Gwenfro Units 4–8, Wrexham Technology Park, Wrexham, LL13 7YP, United Kingdom.

Marie Pirotta, Primary Health Care Research Evaluation and Development Senior Research Fellow, Department of General Practice, 200 Berkeley Street, Carlton Victoria 3053, Australia.

Jeffrey Sisler, Associate Dean, Division of Continuing Professional Development; Professor, Department of Family Medicine, University of Manitoba, 727 McDermot Avenue, Winnipeg, Manitoba, R3E 3P5, Canada.

Berit Skjødeberg Toftegaard, PhD Research Fellow, Research Unit for General Practice, Department of Public Health, Aarhus University, Bartholins Allé 2, 8000 Aarhus C, Denmark.

Associate Professor Hans Thulesius, Associate Professor at Lund University, Box 117, SE-221 00 Lund, Sweden.

Professor Peter Vedsted, Professor at Research Unit for General Practice, Department of Public Health, Aarhus University, Bartholins Allé 2, 8000 Aarhus C, Denmark.

David Weller, James Mackenzie Professor of General Practice, Centre for Population Health Sciences, University of Edinburgh, Doorway 1, Medical Quad Teviot Place, Edinburgh, EH8 9DX, United Kingdom.

Jane Young, Professor in Cancer Epidemiology, Public Health, School of Public Health, D02-QE11 Research Institute for Mothers and Infants, The University of Sydney, 2006, Australia.

## Abbreviations

ICBP: International Cancer Benchmarking Partnership; PCP: Primary care practitioner.

## Competing interests

WH is the clinical lead for the ongoing revision of the NICE 2005 guidance on referral for suspected cancer, CG27. His contribution to this article is in a personal capacity, and is not to be interpreted as representing the view of the Guideline Development Group, or of NICE itself. The other authors declare that they have no competing interests.

## Authors’ contributions

The initial development of the survey in England was undertaken by PWR, WH, CG with support from CF. KA undertook the validation in England. All authors contributed to the development of the initial survey into an international instrument. PWR wrote the original manuscript with subsequent contributions from all authors. PWR, WH, KA, AB, MD, CF, EG, NH, RDN, MP, JS, HT, PV, JY and GR were responsible for ensuring the language and terminology of the survey was relevant to their health economy. PWR, WH, KA, AB, MD, CF, EG, NH, RDN, MP, JS, HT, PV, JY and GR contributed to, reviewed and approved the final manuscript. PV, HT, SSA were responsible for translation into the Scandinavian languages and validation in these countries.

## Pre-publication history

The pre-publication history for this paper can be accessed here:

http://www.biomedcentral.com/1471-2296/15/122/prepub
